# Vegetation and Soil Aggregates Shape Nematode Communities and Energy Flow on the Loess Plateau

**DOI:** 10.3390/microorganisms14040827

**Published:** 2026-04-03

**Authors:** Wenjuan Kang, Zhiming Chen, Yuanyuan Du

**Affiliations:** 1 Key Laboratory of Grassland Ecosystem (Gansu Agricultural University), Ministry of Education, Lanzhou 730070, China; 17309325046@163.com; 2 College of Horticulture, Gansu Agricultural University, Lanzhou 730070, China; chenzm@gsau.edu.cn

**Keywords:** vegetation restoration, soil aggregates, soil nematode, trophic groups, energy flux, energy uniformity, Loess Hilly

## Abstract

Although soil nematodes are central to belowground energy flow, how vegetation and soil aggregate characteristics interactively regulate the nematode community structure and energy dynamics remains poorly understood. We investigated 80 soil samples from five vegetation types—*Prunus armeniaca* L. (AV), *Pinus tabuliformis* Carrière (PT), *Caragana korshinskii* (CK), *Medicago sativa* L. (MS), and native grass *Stipa bungeana* (SB)—and four aggregate sizes (LMA > 2 mm, MMA 0.25–2 mm, SMA 0.053–0.25 mm, and MA < 0.053 mm) on the Loess Plateau. Vegetation types showed clear functional differentiation, in which AV dominated bacterivore diversity and energy flux in LMA, CK enhanced fungivore and herbivore energy flow, SB supported omnivore–carnivore energy flux, and PT exhibited suppressed communities. Fauna analysis of the EI (enrichment index)–SI (structural index) plot revealed aggregate-dependent food web structuring, where all vegetation types clustered in quadrant C (structured, low enrichment) in small aggregates, while PT and MS shifted to quadrant D (structured, enriched) in larger aggregates. SEM showed that energy flux and energy uniformity are driven by nematode abundance (*p* < 0.01) and diversity (*p* < 0.01), respectively, with soil aggregates promoting uniformity (*p* < 0.05) but suppressing total flux (*p* < 0.05), thus revealing a trade-off between energy throughput and distribution equity. CK maximizes total energy flux, while AV maintains high energy uniformity; as such, they could be keystone restoration species in the study area. This study provides mechanistic insights into soil food web energetics and offers an empirical foundation for optimizing vegetation restoration strategies on the Loess Plateau.

## 1. Introduction

The Loess Hilly Region of central Gansu faces severe soil erosion and water scarcity, making it one of China’s most ecologically fragile areas [1,2]. Artificial vegetation restoration plays an important role in improving soil function and controlling soil erosion and, in 1999, the Chinese government launched the “Grain for Green Project” [3] with the objective of restoring the soil ecological environment. Since its commencement, vegetation cover in the Loess Hilly Region has greatly improved. Typical communities of trees, shrubs, grasses, and other species have become established and the ecological environment has shown significant improvement, as vegetation can enhance soil nutrient storage and cycling [4,5]. Through material and energy exchanges with the surrounding environment, different vegetation types alter soil nutrient characteristics based on their biological traits, stand structure, and community features [6], ultimately shaping the ecological environment [7,8].

There are an abundant number of soil nematode species, which are widely distributed across various soil types on Earth [9]. They occupy different trophic levels within the soil micro-food web and play key roles in fundamental processes such as organic matter dynamics and soil aggregate formation. Different soil aggregates offer varied nutrients and spatially heterogeneous habitats for soil nematodes, while rhizosphere soil aggregates serve as the key interface for resource exchange between plants and soil [9,10,11]. Differences in predation pressure and resource availability for various nematode trophic groups across different vegetation types and soil aggregates [12], affecting nematode community structure and metabolic characteristics. Soil nematodes are a key component of the soil metazoan and detritus food web [13] and serve as critical nodes linking energy transfer between soil microorganisms and other soil animals. This connection effectively regulates nutrient flow and cycling [14] and supports biogeochemical cycles in terrestrial ecosystems [15]. The roots of trees, shrubs, and herbs can form symbiotic relationships with soil nematodes and other organisms [16]. In this way, nematodes at different trophic levels can obtain nutrients through parasitism and transfer and accumulate resources through various feeding pathways, ultimately influencing energy flow within the soil micro-food web. Meanwhile, soil nematodes in various trophic functional groups can alter the functionality of the soil micro-food web structure and its role in terrestrial ecosystems by affecting energy flows [17].

In the Loess Hilly Vegetation Restoration Area of central Gansu, the relationship between soil nematodes and carbon flux has been clarified [18], and the energy flow of the soil nematode food web has been calculated using their total metabolic rate from respiration and production [17,19]. Based on these approaches, multiple parameters, including ecological indicators, can be used to assess multifunctional aspects of the soil ecosystem and the effectiveness of vegetation restoration [20,21]. However, to date, few studies have specifically examined the interactive effects of vegetation type and soil aggregate size on soil nematode community structure and energy dynamics in this region. Therefore, this study focuses on the typical arbor, shrub, and grass vegetation types established during artificial vegetation restoration in the Loess Hilly Region of central Gansu, with the objective of clarifying how nematode community characteristics and energy flux patterns respond to vegetation type at the soil aggregate scale, as well as the relationship between nematode community diversity and energy flow. Based on the literature review and ecological theory, we propose the hypothesis that soil aggregates mediate the impacts of vegetation type on the soil nematode community and energy flux. These findings are important for evaluating the micro-food web structure and functionality in soil, as well as for promoting the sustainable development of soil ecosystems.

## 2. Materials and Methods

### 2.1. Experimental Design

#### 2.1.1. General Situation of the Study Area

The experimental site was located in the monitoring area (34°26′–35°35′ N, 103°52′–105°13′ E) of the Dingxi Research Institute of Soil and Water Conservation, Gansu Province, in the Loess Plateau in northwest China, characterized by a semi-arid loess hilly and gully area [22]. The soil type is yellow meadow soil with weak erosion resistance and low organic matter content. The study area is characterized by a temperate continental monsoon climate, with an average altitude of 1900–2250 m. The mean annual temperature is 6.3 °C and the average annual precipitation is 427 mm, while evaporation reaches approximately 1500 mm [23]. Precipitation is predominantly concentrated in summer, primarily occurring as heavy rainfall events.

Prior to the implementation of the “Grain for Green Project”; in 1999, the native grass in the study area was *Stipa bungeana* (SB) grassland. In 1999, apricot forest (*Prunus armeniaca* L., AV), Chinese pine forest (*Pinus tabuliformis* Carrière, PT), and *Caragana korshinskii* (CK) shrublands were artificially established. In 2001, alfalfa grassland (*Medicago sativa* L., MS) was newly planted. No grazing or disturbance occurred in any plots from 1999 to 2023. In July 2023, sample plots were established in areas planted with *P. armeniaca* (AV), *P. tabuliformis* (PT), *C. korshinskii* (CK), and *M. sativa* (MS), from which soil samples were subsequently collected. The control vegetation (SB) has remained unmanaged since 1999 and represents the existing natural grassland community, acting as a baseline for evaluating restoration measures. This vegetation type is characteristic of secondary degradation in the region, which is developed under long-term severe soil erosion.

#### 2.1.2. Sample Collection

In July 2023, during the peak growth season of artificial vegetation in the loess hilly area of Longzhong, soil samples were collected from rhizosphere soil at a depth of 0–30 cm employing a modified 5-point sampling method, with four spatially uniform sampling points arranged [24]. The sampling area was 7 m × 7 m. Four soil samples were obtained from each point, and one composite sample was formed by blending the four individual samples. Each treatment plot had four replicates, with a total sample size of 80 (4 plots × 5 vegetation × 4 aggregates). After eliminating impurities such as stones, gravel, and plant residues from the soil samples, they were thoroughly mixed to form a homogeneous composite sample. The sample was then passed through a 2 mm sieve and promptly preserved on ice. In addition, under relatively sterile conditions, each mixed soil sample was gently separated along its natural texture to preserve aggregate structure. Plant and animal residues, along with small stones, were subsequently removed from the soil using a 5 mm sieve. Each soil sample was partitioned into two aliquots for analysis. The first aliquot was used for the morphological identification of nematodes, while the second aliquot was placed in a dedicated aluminum box for the determination of soil moisture content. The residual soil samples were transported back to the laboratory and air-dried in a shaded area for the measurement of soil physical and chemical parameters.

#### 2.1.3. Soil Aggregate Fractionation

The fresh soil samples (about 500 g) were air-dried to about 15% gravimetric water content and then manually broken to <8 mm. In total, 100 g samples were used for soil aggregate fractionation using a vibrating screening device (STZS-200, DEDU instruments, Changzhou, China) [25]. Briefly, the samples were transferred to a vibrating screening device and sieved at a speed of 500 rpm through various sieve sizes (2 mm, 0.25 mm, and 0.053 mm) for 10 min [26]. Eventually, the soil samples were divided into four soil aggregates with different particle sizes: large macroaggregates (LMA, >2 mm), medium macroaggregates (MMA, 0.25–2 mm), small macroaggregates (SMA, 0.053–0.25 mm), and microaggregates (MA, <0.053 mm). All samples were treated under identical conditions to ensure comparability among treatments.

### 2.2. Morphological Identification of Soil Nematodes

Soil nematodes were extracted using the shallow dish method. A 100 g fresh soil sample was placed on filter paper in a shallow dish [27]. Tap water was slowly added along the inner wall of the dish until a thin water film formed on the soil surface [28]. The sample was then incubated at room temperature for 48 h. After incubation, nematodes were collected by washing the sample through a 500-mesh sieve (pore size: 25 μm) with deionized water. The nematodes were then killed in a 60 °C water bath and subsequently fixed in a 4% formalin solution. Finally, the fixed nematodes were transferred to a pre-labeled specimen vial for identification.

Nematodes were counted under a stereomicroscope (Leica M165C) at 40× magnification. Counts were converted to the number of nematodes per 100 g of dry soil, and nematodes were classified and identified to the genus level based on morphological characteristics [29,30,31]. The online database Nemaplex (http://nemaplex.ucdavis.edu), established by Professor Ferris at the University of California, Davis, was also consulted. Identification was performed using an optical microscope (Olympus CX41) (Olympus Corporation, Tokyo, Japan) at 400× and 1000× magnification. For samples containing more than 100 nematodes, the nematode suspension was scanned systematically row by row under the optical microscope, and the first 100 nematodes encountered were randomly selected and identified without repetition or selective picking to ensure random sampling. For samples with 100 or fewer nematodes, all individuals were identified one by one. Based on the morphological features of the nematode head, feeding guilds, and colonizer–persister values (cp), nematodes were classified into four trophic groups: bacterivores, fungivores, herbivores, and omnivores–carnivores. Nematode cp values were categorized from 1 to 5. The cp 1 group represents a typical r-strategy, characterized by rapid reproduction, a short life cycle, and high dispersal capacity. As the cp value increases, the life-history strategy of nematodes progressively shifts toward a K-strategy. This shift is marked by a decreased reproduction rate, longer generation time, enhanced tolerance to environmental stress, and improved competitiveness [32]. Strict quality control procedures for morphological nematode identification, including repeated examination and consistency checks between multiple observers, were used to ensure the accuracy and reliability of community data.

### 2.3. Determination of Soil Physicochemical Properties

A total of 11 soil physical and chemical parameters were determined; namely, soil moisture, electrical conductivity, pH, soil organic carbon, total nitrogen, nitrate nitrogen, ammonium nitrogen, total phosphorus, available phosphorus, microbial biomass carbon, and microbial biomass nitrogen.

Soil moisture was quantified using the oven drying approach at a temperature of (105 ± 2 °C) with a DHG-9070A oven (Shanghai Jinghong Experimental Equipment Co., Ltd., Shanghai, China). Electrical conductivity was measured using a METTLER TOLEDO FE38 benchtop conductivity meter (METTLER TOLEDO, Greifensee, Switzerland). The pH value was ascertained by the glass electrode method using a pH meter (Model PHSJ-5, INESA Scientific Instrument Co., Ltd., Shanghai, China) with a soil-to-water ratio of 1:2.5. Soil organic carbon was determined via the chromic acid oxidation heating technique [33] using a digital heating block (Model DB-2A, Shanghai Jingke Industrial Co., Ltd., Shanghai, China). Total nitrogen was determined through the Kjeldahl nitrogen determination method using a Kjeldahl nitrogen analyzer (Model KDN-818, Shanghai Shunyu Instrument Co., Ltd., Shanghai, China). Nitrate nitrogen and ammonium nitrogen were assayed via the colorimetric method using a continuous flow analyzer (Model SAN++ System, Skalar, Breda, The Netherlands) [34]. Total phosphorus was determined employing the molybdenum-antimony dichromate colorimetric method [35,36] using a UV–visible spectrophotometer (Model UV-1800, Shimadzu Corporation, Kyoto, Japan). Available phosphorus was measured using the NaHCO_3_ extraction colorimetric method [37] with the aforementioned UV–visible spectrophotometer (Model UV-1800, Shimadzu Corporation, Kyoto, Japan). Microbial biomass carbon and microbial biomass nitrogen were determined by the chloroform fumigation extraction method [34], with extracts analyzed using an elemental analyzer (Model Vario EL III, Elementar Analysensysteme GmbH, Langenselbold, Germany) for microbial biomass carbon and a Kjeldahl nitrogen analyzer (Model KDN-818, Shanghai Shunyu Instrument Co., Ltd., Shanghai, China) for microbial biomass nitrogen.

Quality assurance and quality control (QA/QC) were strictly performed throughout the analysis. Before sample analysis, all indicators were calibrated and validated using certified reference materials (GBW07410, National Research Center for Certified Reference Materials, Beijing, China). Method blanks (distilled water instead of soil samples) were included in each batch analysis to eliminate background contamination and correct potential reagent interference. Ten percent of samples were randomly selected from each index for repeated analysis, and the relative standard deviation (RSD) of repeated measurements was controlled to 5% for re-analysis in order to ensure the reliability of the data.

### 2.4. Energetic Structure of Nematode Communities

#### 2.4.1. Basic Parameters and Data Sources

We used network-wide metrics, including total biomass and total energy flux of trophic groups, to characterize the energetic structure of nematode communities. The average fresh biomass for each taxon was derived from a public database (http://nemaplex.ucdavis.edu/General/WhatsNew.htm, accessed on 14 August 2023). Nematode cp values were applied to standardize the proportion of carbon allocated to production over a unit life course [38].

#### 2.4.2. Carbon Allocated to Production (P_C_)

First, the average biomass of a given taxon was multiplied by its abundance. Carbon allocated to production (P_C_) per individual was calculated assuming a dry weight equivalent to 20% of fresh biomass and a carbon content equivalent to 52% of dry weight [39]. Life-cycle duration (days) was approximated as 12 × cp value [17,30,32].

Total daily production carbon (P_C_) was calculated as:

PC=∑Nt0.1Wtmt12

where N_t_, W_t_, and m_t_ are the number of individuals, the body weight, and the cp values of taxon t, respectively.

#### 2.4.3. Respiration Carbon (R_C_)

Nematode respiration was modeled using the allometric scaling relationship between metabolism and body size [40]. Total carbon used in respiration (R_C_) was calculated as:

$$R_{C}=\sum \left(N_{t}\left(a\left(W_{t}^{b}\right)\right)\right)$$

where b is approximately 0.75 [41], and a is the relative molecular mass ratio of C to O in CO_2_ (12/44 = 0.273). A conversion coefficient of 0.058 was used to estimate daily carbon respiration in μg [42].

#### 2.4.4. Total Energy Flux (F)

The total energy flux of nematodes (F, μg C 100 g^−1^ dry soil day^−1^) was calculated as the sum of the production and respiration [17,38], with N_t_, W_t_, and m_t_ defined as above.

F=∑Nt0.1Wtmt12+0.273×0.058Wt0.75

#### 2.4.5. Potential Carbon Flux (F_i_) and Energy Loss (L)

The carbon flux into each node was determined using assimilation efficiency (e_a_) and energy loss (L) to higher trophic levels [18,43,44].

Fi=F+Lea

Here, F_i_ represents the potential carbon flux derived from the biomass of nematode trophic group i.

Assimilation efficiencies for nematodes, defined as the fraction of ingested food allocated to respiration and production [44], were set based on the established literature [43,45]: 0.25 for herbivores, 0.60 for bacterivores, 0.38 for fungivores, and 0.5 for omnivores–carnivores.

Energy loss from lower trophic groups to omnivores–carnivores was calculated as:

$$L=D_{io}\times F_{o}$$

where F_o_ represents nematode energetic demands, and D_io_ represents the density-dependent feeding preference of omnivores–carnivores for trophic group i, which was assigned based on the relative proportional abundance of each prey trophic group in the community [18]. Due to the lack of species-specific feeding preference data for the nematode communities in our study, we adopted a uniform feeding preference as a common and conservative simplification when detailed dietary data were unavailable [18].

#### 2.4.6. Energy Flow Uniformity

The flow uniformity of energy in the nematode food web was calculated as the ratio of the mean summed energy flux across channels to the standard deviation of these mean values [46]. This index reflects the evenness of energy distribution among channels.

**Figure 1:**
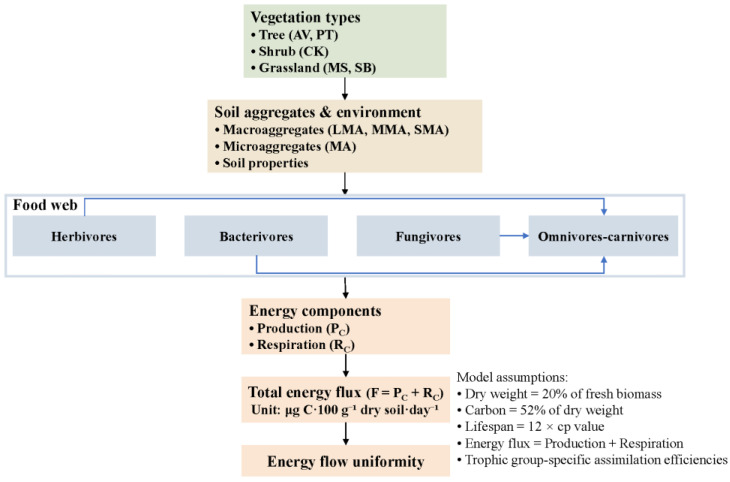
Conceptual diagram of soil nematode trophic relationships and energy flux pathways under different vegetation types and soil aggregates. Arrows indicate the direction of energy flow within the micro-food web. Key parameters and assumptions used in the energy flux calculation are also presented.

In addition, a conceptual diagram (Figure 1) is provided to clearly show the connection between each calculation link and the soil food web, where all model assumptions are explicitly stated and explained.

### 2.5. Data Analysis

#### 2.5.1. The Ecological Function Index of the Soil Nematode Community

The micro-food web structure, nutrient enrichment conditions, and decomposition pathways of the soil ecosystem were assessed using various nematode-based indicators [47]. These indicators include the basic index and maturity index of free-living nematodes [32], the plant-parasitic nematode maturity index [32], the channel index, structural index (SI), and enrichment index (EI) [48,49].

#### 2.5.2. The Metabolic Footprint of the Soil Nematode Community

The metabolic footprint of nematodes (NMF) was determined by calculating the fresh weight of nematodes, as outlined in the Nematodes-Plant Expert Information System.

NMF=∑Nt×0.1×Wtmt+0.273Wt0.75

In this formula, N_t_ is 1, which denotes the abundance of nematodes affiliated with trophic group t, m_t_ is the associated cp value, and W_t_ is the biomass [38].

The metabolic footprint of nematode nutrient groups includes the metabolic footprint of bacterivores, fungivores, herbivores, and omnivores–carnivores. The first three categories represent the carbon and energy input from bacteria, fungi, and plants into the food web, respectively, while the latter category indicates the carbon and energy input into the higher trophic level of omnivorous–carnivores. The total nematode metabolic footprint quantifies the overall metabolic footprint of nematode populations [38].

#### 2.5.3. Soil Nematode Faunal Analysis

Enrichment metabolic footprint (*Fe*) and structural metabolic footprint (*Fs*) metrics were utilized for the analysis of the nematode fauna. The enrichment footprint (*Fe*) represents the metabolic footprint of nematode populations featuring low cp values (1–2), which can rapidly respond to resource accumulation. The structure footprint (*Fs*) reflects the metabolic footprint of nematodes with a high cp value (3–5) [38,50]. The EI and SI thresholds (EI = 50, SI = 50) were used to define quadrant boundaries [38,47]. By taking the coordinate point (SI, EI) as the central position, the coordinate positions (SI − 0.5*Fs*/k, EI), (SI + 0.5*Fs*/k, EI), (SI, EI − 0.5*Fe*/k), and (SI, EI + 0.5*Fe*/k) of each treatment in four quadrants were determined, where k denotes the conversion factor [51].

#### 2.5.4. Structural Equation Model

A structural equation model was constructed by employing the lavaan package (version 0.6–18) of the R software version 4.4.1, in order to elucidate the potential relationship among soil aggregates and soil environment, soil nutrition (carbon, nitrogen, phosphorus), nematode abundance, nematode diversity, total energy flow, and energy flow uniformity. Before modeling, principal component analysis was conducted on four groups of variables, excluding single indices: (1) soil environmental factors (soil moisture, electrical conductivity, and pH); (2) soil carbon nutrients (soil organic carbon and microbial biomass carbon); (3) soil nitrogen nutrients (total nitrogen, nitrate nitrogen, ammonium nitrogen, and microbial biomass nitrogen); and (4) soil phosphorus nutrients (total phosphorus and available phosphorus). The first principal component from each group was then extracted for modeling [52].

Prior to SEM, all observed variables were standardized to a mean of 0 and a standard deviation of 1 to ensure consistent variable scaling and facilitate the interpretation and comparison of path coefficients across different variables. Data transformation was performed to meet the assumptions of SEM: normality and homoscedasticity of data were first verified using a Shapiro–Wilk test and Levene’s test, respectively. Variables that violated these assumptions were log-transformed to improve data normality and reduce heteroscedasticity, ensuring the reliability of model estimates. Missing data handling was conducted using the full information maximum likelihood (FIML) estimator. The proportion of missing values in the dataset was less than 5%, and missingness was determined to be missing at random (MAR) based on preliminary analysis. Arrows and their associated path coefficients indicate the direction and strength of the relationships among these variables. The optimal model was determined through the iterative elimination of non-essential paths and reducing redundant parameters. The SEM model was fitted using the FIML estimator, which is robust to mild violations of multivariate normality. Model fitness was evaluated using multiple complementary indices: the chi-square (χ^2^) statistic and its corresponding *p*-value, degrees of freedom (df), comparative fit index (CFI), goodness-of-fit index (GFI), incremental fit index (IFI), root mean square error of approximation (RMSEA), and standardized root mean square residual (SRMR). In general, when χ^2^/df < 3; *p* > 0.05; CFI, GFI, and IFI values are closer to 1; RMSEA < 0.05; and SRMR < 0.08, the model fits better.

#### 2.5.5. Alpha and Beta Diversity

The α-diversity of nematode communities was evaluated using the Shannon index [53]. Non-metric multidimensional scaling (NMDS) based on the Bray–Curtis distance was performed to visualize beta diversity patterns among vegetation types and soil aggregates [54]. Permutational multivariate analysis of variance (PERMANOVA) (F and *p*) was applied to test the significance of grouping factors. Analyses were conducted using Wekemo Bioincloud (https://www.bioincloud.tech/) [54]. A stress value was used to evaluate the reliability of the NMDS ranking results, and the general requirement was <0.2.

Prism 8.0.2 was used for analysis of the relationships between nematode diversity and total energy flux or flux uniformity of nematode communities through linear regression. Statistical analyses and data visualization were also conducted using Prism 8.0.2. A two-way analysis of variance (ANOVA) was performed. Differences between groups were analyzed via Tukey’s HSD test for multiple comparisons (*p* = 0.05) and a post hoc test. Before the ANOVA analyses, the data were checked for normality (Shapiro–Wilk test) and homoscedasticity (Levene’s test), and variables that violated these assumptions were logarithmically converted. Data are presented as the mean ± standard error (SE). The sample size for each treatment was *n* = 4.

## 3. Results

### 3.1. Alpha and Beta Diversity of Soil Nematodes

**Figure 2:**
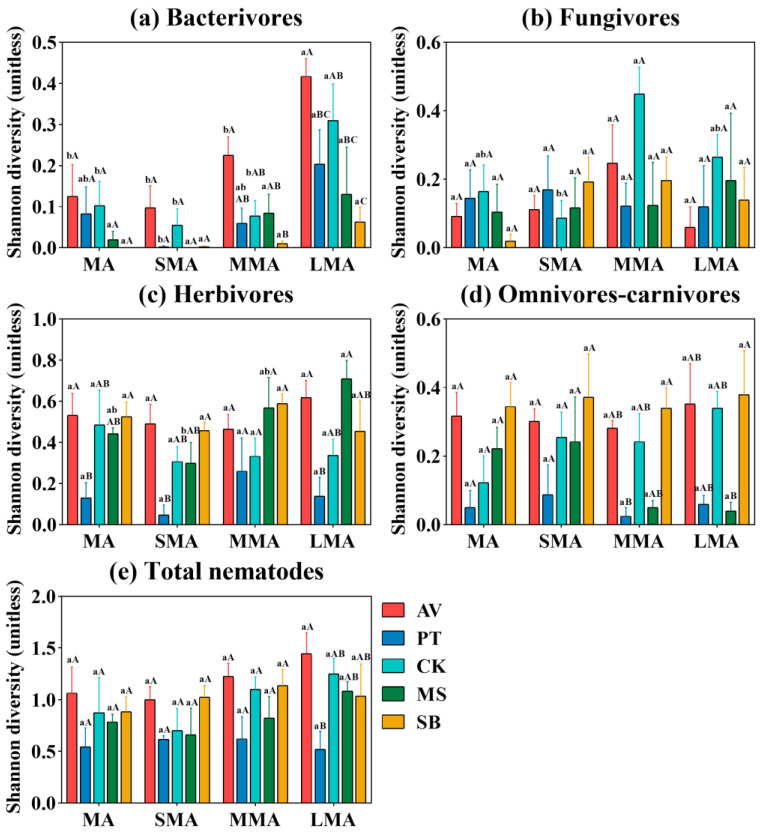
Shannon diversity index of nematode trophic groups (**a**–**d**) and total nematodes (**e**) under different soil aggregates. MA, SMA, MMA, and LMA are aggregates with different grain size, as detailed in Section 2. Lowercase letters denote significant differences among different grain sizes for the same vegetation type (*p* < 0.05), while uppercase letters denote significant differences between various vegetation types within the same grain size (*p* < 0.05). AV, *Prunus armeniaca* L.; PT, *Pinus tabuliformis* Carrière; CK, *Caragana korshinskii*; MS, *Medicago sativa* L.; SB, *Stipa bungeana*.

In this study, the Shannon index is used to represent the α-diversity. The effects of different vegetation types and soil aggregate size on the diversity of nematodes and total nematodes showed obvious differentiation (Figure 2). LMA is more conducive to maintaining a high diversity of most nematode trophic groups, among which bacterivores are the most prominent. The nematode diversity of PT was generally low. AV was dominant in bacterivores (Figure 2a) and herbivores (Figure 2c). CK and SB were dominant in fungivores (Figure 2b) and omnivores–carnivores (Figure 2d), respectively. There was no significant difference in the diversity of total nematodes among soil aggregates of different vegetation types (*p* > 0.05), and only LMA showed vegetation differences (Figure 2e).

**Figure 3:**
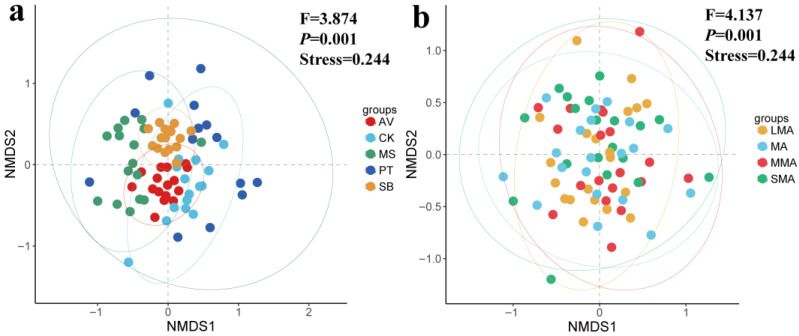
Non-metric multidimensional scaling (NMDS) based on the Bray–Curtis distance showed beta diversity patterns among vegetation types (**a**) and soil aggregates (**b**). A permutational multivariate analysis of variance (PERMANOVA) (F and *p*) was applied to test the significance of grouping factors. A stress value was used to evaluate the reliability of NMDS ranking results, with 0.244 indicating an acceptable ordination reliability. MA, SMA, MMA, and LMA are aggregates with different grain size, as detailed in Section 2. AV, *Prunus armeniaca* L.; PT, *Pinus tabuliformis* Carrière; CK, *Caragana korshinskii*; MS, *Medicago sativa* L.; SB, *Stipa bungeana*.

The NMDS plot was used to present the beta diversity, which revealed a clear separation among the vegetation types (Figure 3a) and soil aggregates (Figure 3b). A PERMANOVA confirmed that there were significant differences in nematode composition among vegetation types (F = 3.874, *p* = 0.001) and soil aggregates (F = 4.137, *p* = 0.001). The stress value (0.244) indicated an acceptable ordination reliability (Figure 3a,b). AV formed a distinct cluster in the negative NMDS1 region, while the similarity of nematode communities in CK was higher (Figure 3a). The distribution pattern of four soil aggregates in the NMDS space reflects that the nematode community structure had group specificity (Figure 3b). The sample points of MA were scattered; other groups were relatively concentrated. The spatial distance between MMA and LMA was the farthest.

### 3.2. Community Composition of Soil Nematodes

A total of 109,488 nematodes were identified based on morphology, with an average density of 1369/100 g dry soil (Supplementary Figure S1). They comprised 2 classes, 8 orders, 9 families, and 15 genera (Supplementary Table S1). Among soil aggregates, the number of nematodes showed a particle size gradient of MMA > LMA > SMA > MA. Among the vegetation types, the number of nematodes in SB was the highest, which was significantly higher than that in other vegetation types (*p* < 0.05), followed by CK, while MS was the lowest (Supplementary Figure S1). With an increase in soil aggregate size, the number of nematodes showed a downward trend (Supplementary Table S1). The dominant nematode groups among aggregates significantly differed, where MMA was more conducive to a higher number of nematodes and enrichment of herbivores, while LMA was more supportive of omnivores–carnivores. CK and AV helped to increase the number of nematodes, with fungivores being the most abundant in the four aggregate sizes, while omnivores–carnivores were the most abundant in small aggregate sizes (MA, SMA). The number of PT nematodes was generally low; SB had more omnivores–carnivores under large aggregate sizes (MMA and LMA).

### 3.3. Energy Metabolic Footprint of Soil Nematodes

**Figure 4:**
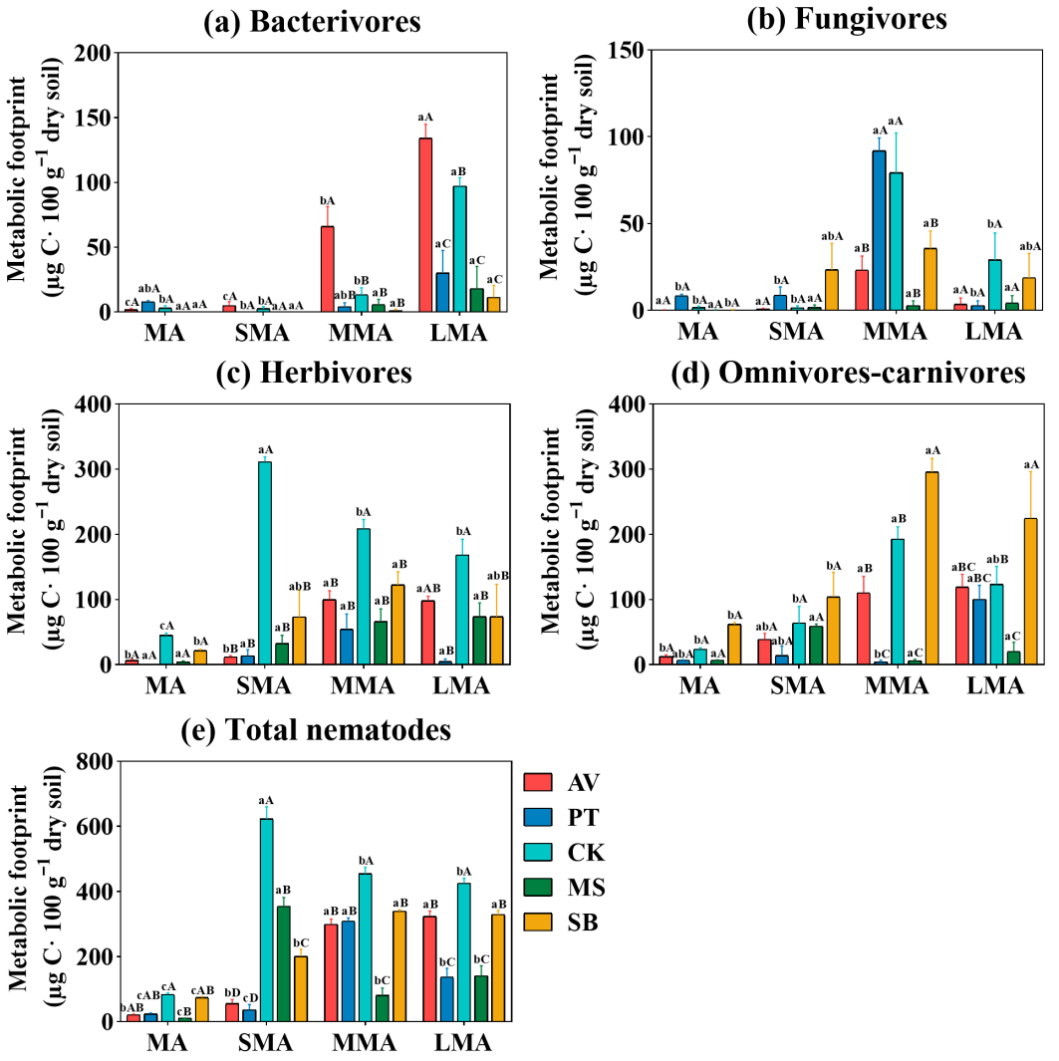
Metabolic footprint of nematode trophic groups (**a**–**d**) and total nematodes (**e**) under different soil aggregates. MA, SMA, MMA, and LMA are aggregates with different grain size, as detailed in Section 2. Lowercase letters denote significant differences among different grain sizes for the same vegetation type (*p* < 0.05), while uppercase letters denote significant differences between various vegetation types within the same grain size (*p* < 0.05). AV, *Prunus armeniaca* L.; PT, *Pinus tabuliformis* Carrière; CK, *Caragana korshinskii*; MS, *Medicago sativa* L.; SB, *Stipa bungeana*.

The energy metabolic footprints of nematodes in each trophic group and total nematodes were affected by vegetation type and soil aggregate size. The energy metabolic footprints were significantly different among trophic groups, and the dominant pattern was clear (Figure 4). Bacterivores are the core of energy metabolism in AV (Figure 4a), and total metabolism is the strongest in LMA. PT is characterized by the energy metabolism of fungivores (Figure 4b). CK has an absolute advantage in driving herbivores (Figure 4c) and the total nematode metabolic footprint, while SB has a supporting role in the high metabolism of omnivores–carnivores (Figure 4d); the metabolic characteristics of MS have no obvious regularity. MMA/SMA are more conducive to the metabolism of herbivores, fungivores, and omnivores–carnivores, while LMA is more conducive to bacterivore metabolism and the total energy metabolism of AV. The energy metabolic footprint of total nematodes showed that the aggregate size matched the vegetation type (Figure 4e); that is, CK and MS peaked in SMA, PT and SB peaked in MMA, and AV peaked in LMA.

### 3.4. Ecological Function Index of Nematode Communities

**Figure 5:**
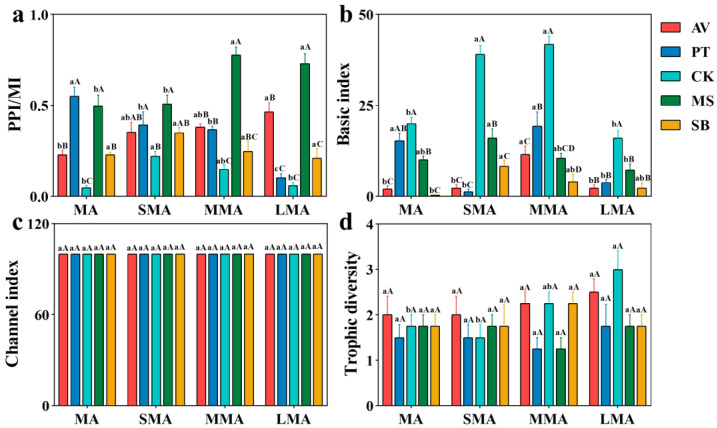
Functional structure index of soil nematodes under different soil aggregates. (**a**) The ratio of plant-parasitic nematode maturity index (PPI) to maturity index (MI); (**b**) Basic index; (**c**) Channel index; (**d**) Trophic diversity. MA, SMA, MMA, and LMA are aggregates with different grain size, as detailed in Section 2. Lowercase letters denote significant differences among different grain sizes for the same vegetation type (*p* < 0.05), while uppercase letters denote significant differences between various vegetation types within the same grain size (*p* < 0.05). AV, *Prunus armeniaca* L.; PT, *Pinus tabuliformis* Carrière; CK, *Caragana korshinskii*; MS, *Medicago sativa* L.; SB, *Stipa bungeana*.

The analysis of the nematode community ecological function index showed that the PPI/MI (Figure 5a) and BI (Figure 5b) were more sensitive to vegetation types and soil aggregate sizes. Specifically, CK had a high BI and low PPI/MI, MS had a high PPI/MI, and MMA was more conducive to maintaining a high BI. Comparatively, CI (Figure 5c) and TD (Figure 5d) were relatively stable with no obvious difference.

### 3.5. Soil Nematode Fauna Analysis

**Figure 6:**
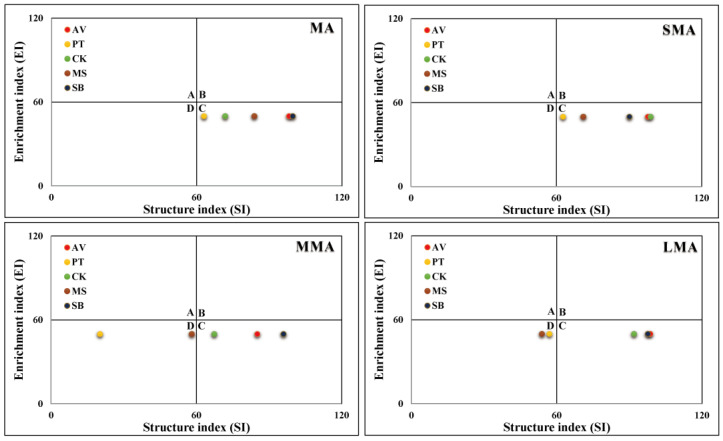
Fauna analysis of soil nematodes under different soil aggregates. A, B, C, and D represent four quadrants, respectively. MA, SMA, MMA, and LMA are aggregates with different grain size, as detailed in Section 2. AV, *Prunus armeniaca* L.; PT, *Pinus tabuliformis* Carrière; CK, *Caragana korshinskii*; MS, *Medicago sativa* L.; SB, *Stipa bungeana*.

The nematode fauna analysis (Figure 6) showed that soil aggregate size was the key factor affecting the distribution pattern of nematode fauna. In MA and SMA, the five vegetation types are concentrated in the C quadrant; in MMA and LMA, the flora are differentiated, PT and MS are transferred to the D quadrant, and AV, CK, and SB are still stably distributed in the C quadrant. This shows that an increase in aggregate size drives differentiation of the nematode fauna distribution. PT and MS are more sensitive to a change in aggregate size, while the nematode fauna structure of AV, CK, and SB is more stable.

### 3.6. Energy Flow of Soil Nematodes

**Figure 7:**
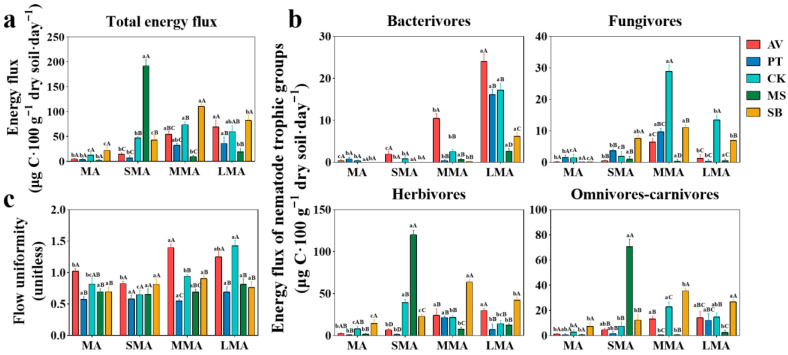
The energy flux of total nematodes (**a**), nematode trophic groups (**b**), and flow uniformity (**c**) under different soil aggregates. MA, SMA, MMA, and LMA are aggregates with different grain size, as detailed in Section 2. Lowercase letters denote significant differences among different grain sizes for the same vegetation type (*p* < 0.05), while uppercase letters denote significant differences between various vegetation types within the same grain size (*p* < 0.05). AV, *Prunus armeniaca* L.; PT, *Pinus tabuliformis* Carrière; CK, *Caragana korshinskii*; MS, *Medicago sativa* L.; SB, *Stipa bungeana*.

The energy flow and energy uniformity of soil nematodes were regulated by vegetation type and soil aggregate size, and nematodes of different trophic groups showed clear response patterns (Figure 7). The energy flow of total nematodes was the lowest in MA and increased with aggregate size. There was obvious vegetation differentiation; that is, AV and PT were the highest in LMA, and CK and SB were the highest in MMA (Figure 7a). In terms of the energy flow of nematodes in each trophic group (Figure 7b), the energy flow of bacterivores in the four non-MS vegetation types was the highest in LMA, and AV was significantly higher than CK (*p* < 0.05). Fungivores were the highest in MMA, and CK was higher than SB and MS (*p* < 0.05). Herbivores were the lowest in MA, and SB was the highest in all soil aggregates, which was significantly higher than PT (*p* < 0.05). There were differences in the aggregate size of omnivores–carnivores in all vegetation types, and MMA was significantly higher than MA. The energy flow of SB omnivores–carnivores was the highest in MMA and LMA. In terms of energy uniformity (Figure 7c), AV had the highest energy uniformity in MA and MMA, while CK had the highest energy uniformity in LMA, followed by AV.

In summary, MA is generally the trough of the nematode energy flow, and MMA and LMA are the energy peaks. SB drives the high energy flow of herbivores and omnivores–carnivores, AV supports bacterivore energy flow and energy uniformity, and CK enriches fungivores and maintains high energy uniformity under large-size aggregates.

**Figure 8:**
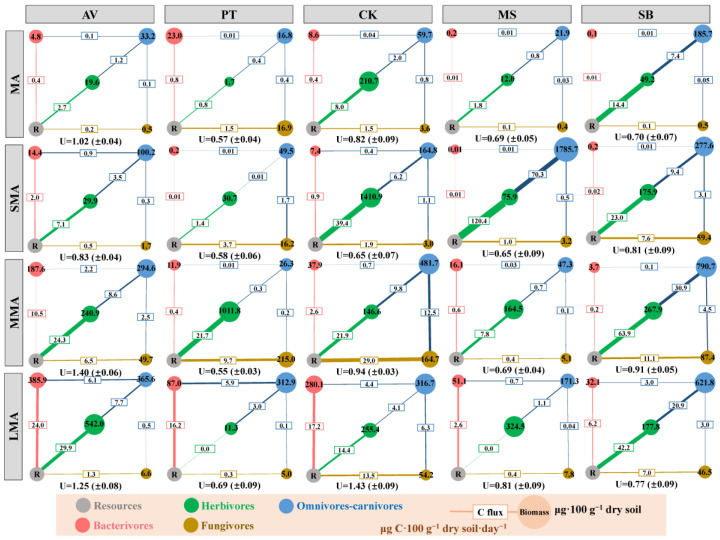
The energetic structures of soil nematodes under different soil aggregates. For each soil aggregate, a five-node food web was constructed with bacterivores (red), fungivores (brown), and herbivores (green) receiving energy from basal resources (R, gray), and omnivores–carnivores (blue) receiving energy from other nodes. Numbers along the lines represent energy flux (μg C·100 g^−1^ dry soil·day^−1^). The size of nodes corresponds to the amount of fresh biomass (μg·100 g^−1^ dry soil). The uniformity (U) of soil nematode energetic structure (unitless, mean ± standard error) was calculated as the ratio of the mean of summed energy flux through each energy channel to the standard deviation of these mean values. Prism 8.0.2 was used to conduct a two-way analysis of variance (ANOVA) of energy flux, uniformity (U), and biomass. MA, SMA, MMA, and LMA are aggregates with different grain size, as detailed in Section 2. AV, *Prunus armeniaca* L.; PT, *Pinus tabuliformis* Carrière; CK, *Caragana korshinskii*; MS, *Medicago sativa* L.; SB, *Stipa bungeana*.

The biomass and energy channel pattern of nematodes changed significantly with soil aggregate sizes and showed stable vegetation differentiation (Figure 8). In terms of the transformation of biomass structure driven by soil aggregate size, in MA and SMA, CK was dominated by herbivore biomass, while AV, MS, and SB were dominated by omnivore-carnivore biomass. In MMA and LMA, the biomass of omnivores–carnivores was dominant in CK and SB, while the biomass of herbivores was the highest in MS; the biomass of omnivores–carnivores in AV and herbivores in PT peaked at two soil aggregate sizes, respectively.

In contrast, vegetation type plays a regulatory role in energy channels. Compared to SB, CK increased bacterivore and herbivore biomass, strengthening energy channels from resources to bacterivores and herbivores. AV enhanced energy transfer from resources to bacterivores and from lower to higher trophic levels. In MMA and LMA, CK and PT enhanced resource flow to bacterivores and upward trophic transfer, while AV and MS increased bacterivore biomass. Regarding energy uniformity, AV and CK exhibited higher energy uniformity, particularly in MMA and LMA aggregates.

The above results (Figure 8) showed that with an increase in soil aggregate size, the dominant nematode biomass changed from “differentiation of herbivores and omnivores–carnivores” to “omnivores–carnivores generally dominant”. CK and AV significantly improved energy flux and energy uniformity and were the key vegetation to regulate soil food network structure and energy distribution.

### 3.7. Relationship Between Nematode Communities, Energy Flow, and Soil Environment Across Aggregates

**Figure 9:**
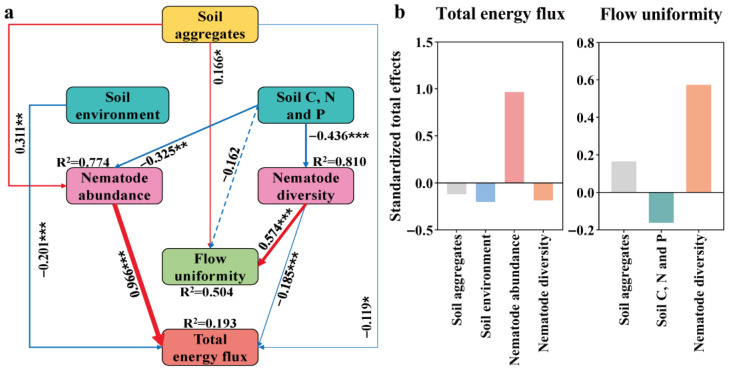
The relationship between soil aggregate, nematode community, energy flux, and soil environment. (**a**) represents the relationship between soil aggregates and soil environment, soil nutrition [C (carbon nutrients), N (nitrogen nutrients), P (phosphorus nutrients)], nematode abundance, nematode diversity, total energy flow, and energy flow uniformity. The lavaan package of the R software version 4.4.0 was employed to construct the structural equation model (X^2^ = 10.678, df = 7.000, *p* = 0.153, CFI = 0.983, GFI = 0.945, IFI = 0.984, RMSEA = 0.081, and SRMR = 0.071). Positive and negative paths are marked with red and blue arrows, respectively. In contrast, significant (marked by * *p* < 0.05, ** *p* < 0.01, *** *p* < 0.001) and non-significant links are represented by solid and dashed arrows, respectively. The width of the lines represents the standardized regression weights. R^2^ values adjacent to the variables indicate the proportion of variance explained by the other variables. (**b**) represents the standardized total effects of each factor on total energy flow and flow uniformity.

The structural equation model showed that soil aggregates, soil environment, and nematode communities formed an energy regulation network (Figure 9a). Soil aggregates significantly increased nematode abundance (*p* < 0.01) and energy uniformity (*p* < 0.05), while inhibiting total energy flow (*p* < 0.05). Soil C, N, and P could significantly reduce the abundance and diversity of nematodes (*p* < 0.01), but had no significant negative effect on energy uniformity. Nematode abundance was the core positive driving factor of total energy flow (*p* < 0.01), while nematode diversity dominated the improvement of energy uniformity while inhibiting total energy flow (*p* < 0.01). Soil environmental factors significantly negatively regulated the total energy flow (*p* < 0.01) (Figure 9b).

In summary, total energy flux and energy uniformity were directly and dominantly driven by nematode abundance and diversity, respectively. Soil aggregates achieved bidirectional regulation of energy processes through direct effects and mediation effects centered on nematode communities; namely, promoting energy distribution uniformity while suppressing total energy flux, ultimately forming a trade-off pattern between total energy throughput and distributional equity.

**Figure 10:**
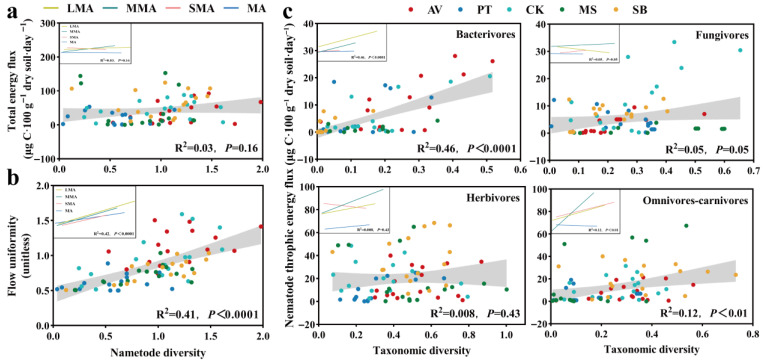
Relationships between the nematode diversity and total energy flux (**a**) or flux uniformity (**b**) of nematode communities and between the diversity and corresponding energy flux of nematode trophic groups (**c**). Linear regression analysis between the nematode diversity and total energy flux or flux uniformity of nematode communities was conducted using Prism 8.0.2. R^2^ values adjacent to the variables indicate the proportion of variance explained by the other variables. Significance levels are indicated by *p*-values. The gray shaded area represents the 95% confidence interval for the regression line. MA, SMA, MMA, and LMA are aggregates with different grain size, as detailed in Section 2. AV, *Prunus armeniaca* L.; PT, *Pinus tabuliformis* Carrière; CK, *Caragana korshinskii*; MS, *Medicago sativa* L.; SB, *Stipa bungeana*.

Regression analysis (Figure 10) showed that the association between nematode diversity and energy process had obvious nematode trophic group dependence and vegetation specificity. At the nematode community level, there was no significant correlation between total nematode diversity and energy flow (Figure 10a), but there was a significant positive correlation with energy uniformity (*p* < 0.01) (Figure 10b), indicating that energy uniformity was consistently driven by total diversity.

At the level of trophic group (Figure 10c), the energy flow showed a pairing response between nematode trophic groups and vegetation types. The diversity of bacterivores was significantly positively correlated with energy flow in vegetation types except for PT and soil aggregates (*p* < 0.01). The diversity of fungivores was positively correlated with energy flow in CK, AV, and MS (*p* < 0.05) and negatively correlated in PT (*p* < 0.05). Herbivores showed a significant positive correlation between energy flow and diversity only in PT (*p* < 0.01). The diversity of omnivores–carnivores was significantly positively correlated with energy flow only in MS (*p* < 0.01) and was strongly regulated by aggregate size. There was a significant negative correlation in MA (*p* < 0.01) and positive correlations in other aggregate sizes.

## 4. Discussion

This study investigated the responses of nematode community structure, diversity, metabolic footprint, and energy flow characteristics to five vegetation types and four soil aggregate fractions. We identified the following key findings: (1) vegetation type shapes nematode community composition; (2) soil aggregate size acts as a microhabitat filter; (3) vegetation and aggregate interactively affect energy flux; (4) fauna analysis showed that food web structure was soil aggregate-dependent and had specific sensitivity among vegetation types (PT/MS shifting; AV/CK/SB stable); (5) energy flux and uniformity are differentially regulated by abundance and diversity; and (6) diversity–energy relationships are trophic group- and vegetation-specific. These results strongly support the central hypothesis that soil aggregates mediate the effects of vegetation on nematode communities and energy flow, while revealing unexpected vegetation-specific responses in fauna analysis and diversity–energy relationships.

### 4.1. Vegetation-Specific Mechanisms Driving Nematode Trophic Group Differentiation

In this study, AV showed the highest bacterivore diversity and energy flux, especially in LMA, and has a unique nematode community structure, which is most differentiated from other vegetation types. This pattern can be mechanistically linked to three interrelated factors. First, AV produces high-carbon litter with elevated C:N ratios, which selectively favors bacterial-based decomposition channels as bacteria are more efficient at utilizing labile carbon compared to fungi [5,55]. Second, as an arbuscular mycorrhizal (AM) tree species, AV root exudates are dominated by simple sugars and organic acids that directly stimulate bacterial growth in the rhizosphere [56,57]. Third, the physical structure of LMA—which accumulate fresh particulate organic matter and maintain high pore connectivity—provides an optimal habitat for bacterivores that require access to bacterial biofilms [10,58]. The positive correlation between bacterivore diversity and energy flux across all vegetation types (except PT) confirms that bacterivore-mediated energy channels are diversity-dependent, with higher species richness enhancing resource exploitation efficiency [59,60].

CK demonstrated the highest fungivore diversity and dominated herbivore energy flux and total metabolic footprint, with a high within-group similarity. As a leguminous shrub, the biological nitrogen fixation ability of CK enriches soils with nitrogen, which helps in lowering litter C:N ratios and shifting decomposition pathways toward the fungal-mediated processing of more recalcitrant materials [61,62]. At the same time, CK develops extensive root systems with high turnover rates [63,64], providing continuous carbon inputs that sustain fungal networks and create infection sites for herbivores [65,66,67]. The positive correlation between fungivore diversity and energy flux in CK indicates that fungivore-mediated energy channels are also diversity-dependent in this nitrogen-rich environment. CK can also promote homogeneous nematode community assembly, potentially through consistent rhizosphere effects mediated by root exudates rich in organic acids and flavonoids that attract specific microbial symbionts [10,19,57,67]. Furthermore, CK’s ability to maintain high energy uniformity in LMA and MMA suggests that nitrogen-fixing plants promote equitable energy distribution across trophic levels, potentially through enhanced resource quality that supports multiple consumer groups simultaneously [68,69].

PT consistently exhibited low nematode diversity across all trophic groups and minimal energy flux. This suppression may be related to several conifer-specific mechanisms. First, Pinus species like PT can produce recalcitrant litter with high lignin content and low nitrogen [70,71,72,73], which limits resource availability for bacterial and fungal decomposers and, consequently, their nematode grazers [74,75,76]. Second, coniferous forests are associated with ectomycorrhizal (ECM) fungi, which can compete with free-living microbes for nutrients, potentially reducing prey availability for bacterivores and fungivores [77,78]. Third, Pinus root exudates contain phenolic compounds and terpenes possessing allelopathic properties, which may directly inhibit nematode survival or indirectly affect them by suppressing microbial prey [16,79,80]. The negative correlation between fungivore diversity and energy flux in PT is particularly striking, suggesting that increased fungivore diversity in coniferous systems may reflect resource partitioning under limited conditions rather than enhanced energy flow.

SB is the native grass species in the study area which exhibited the highest omnivore–carnivore diversity and energy flux, particularly in larger aggregates. This may be associated with the complex and multi-channel food web structure of perennial grasslands. Stipa roots form continuous pore networks, which are conducive to the movement of predators and the entry of prey across soil aggregates [81], and different root exudate profiles support the energy channels of various microorganisms, thereby maintaining the biomass of high-trophic omnivores–carnivores [14]. The positive correlation between the diversity and energy flux of omnivores–carnivores in MS showed that the predator–prey interaction in grassland was diversity-dependent, and higher predator richness enhanced top-down control and energy transfer efficiency [82]. It is worth noting that this kind of relationship was different in MA (negative) and larger aggregates (positive), indicating that microaggregates impose physical constraints on predator–prey interactions, which are alleviated in more connected pore networks [9].

### 4.2. Soil Aggregates Drive Nematode Community Assembly and Energy Dynamics in the Form of Microhabitat Filters

The nematode abundance result (MMA > LMA > SMA > MA) and the maximum dissimilarity between MMA and LMA, as well as the scattered distribution of MA in NMDS ordination, provide strong evidence for the role of soil aggregate size, which acts as a powerful microhabitat filter through regulation of habitat accessibility, resource quality and acquisition, and microenvironmental stability.

Different sizes of aggregate structure impose different degrees of constraint on the movement of nematodes and the acquisition of resources [11,14]. MMA (0.25–2 mm) optimally balances connectivity and conservation, as its intermediate pore network promotes migration while providing a predatory refuge [83,84], which explains the highest abundance of nematodes in MMA. On the contrary, the narrow pore neck in MA (<0.053 mm) causes physical isolation and limits the accessibility of prey, which is the reason for the persistent low energy flux in all vegetation types [85]. The size of soil aggregates was also related to the composition of organic matter and carbon dynamics [10,12]. LMA (>2 mm) is rich in fresh, easily degradable carbon [10], which supports the higher bacterivore diversity and bacterivore-dominated energy fluxes found in this study. MMA contains a mixture of organic matter that maintains both bacterial and fungal channels [12], which lays the foundation for their use as energy hotspots for fungivores and omnivores–carnivores. The richness of nematodes decreased with soil aggregate size in this study, indicating that smaller aggregates with higher heterogeneity supported more genera, while larger aggregates tended to display competitive dominant species [11,86]. Moreover, the enrichment of omnivores–carnivores in LMA may be related to its high stability, which reduced the disturbance mortality of higher trophic nematodes [32].

### 4.3. Fauna Analysis Reveals Aggregate-Dependent Food Web Structuring

This study found that, in MA and SMA (0.053–0.25 mm), all vegetation types were clustered in the C quadrant (structured food webs with low enrichment). This indicates that the stable and resource-limited microhabitats in small aggregates and the physical protection of organic matter limit the activity of microorganisms and the reproduction rate of nematodes [44]. The shift in PT and MS in MMA and LMA to the D quadrant (structured, enriched food webs) represents a fundamental change in food web conditions. For PT, this may be caused by the increase in fungal biomass and hyphal networks in larger soil aggregates that contribute to resource retention and maintain a continuous energy channel [87,88]. For MS, this may be due to the presence of a rhizosphere priming effect in larger aggregates with concentrated root exudates, which stimulates microbial activity without destroying the food web structure [89,90]. On the contrary, AV, CK, and SB were stable in quadrant C at all aggregate scales, indicating that these vegetation types maintained a structured food web and their rhizosphere-associated nematode communities were intrinsically stable regardless of habitat heterogeneity at the aggregate scale [49].

### 4.4. SEM Reveals Dual Pathways Regulating Energy Flux and Uniformity

This study found that energy flux and uniformity are driven by different components of the nematode community; in particular, the total energy flux is mainly determined by nematode abundance (path coefficient is positive, *p* < 0.01), while energy uniformity is positively regulated by nematode diversity (*p* < 0.01) and negatively regulated by abundance (although not significantly). This functional differentiation indicates that higher species richness can ensure the consistency of cross-trophic resource utilization through complementary resource utilization and response diversity [18,46]. The strong positive correlation between total diversity and energy uniformity under all treatments further confirmed this relationship. These results are consistent with the recent meta-analysis which found that biodiversity improves the uniformity of energy distribution in multitrophic systems [68,69]. Meanwhile, we found that soil aggregates had a dual regulatory effect on energy flux and energy uniformity; that is, aggregates directly increased nematode abundance (*p* < 0.01) and energy uniformity (*p* < 0.05), while inhibiting total energy flux (*p* < 0.05). This indicates that there is a trade-off between energy throughput and distribution fairness. Larger aggregates have well-connected pore networks, which can promote energy flow to multiple consumer groups and enhance their uniformity, while imposing physical constraints to limit the maximum flux that can be achieved [11]. In addition, the inherent resource heterogeneity in the structural aggregates may cause energy distribution between multiple channels, reducing the dominance of any single trophic group of nematodes, thereby reducing the total flux and improving uniformity [14]. The negative effects of soil C, N, and P on nematode abundance and diversity (*p* < 0.01) may reflect a nutrient saturation effect; that is, high resource availability is beneficial to microbial competitors rather than nematodes, or reduces habitat stability by promoting rapid decomposition [21,36].

### 4.5. Multidirectional Correlations Between Diversity and Energy Flow Is Trophic-Dependent and Vegetation-Specific

The regression analysis in this study showed that the relationship between diversity and energy flow was not unidirectional but had trophic group-dependent and vegetation-specific multidirectionality, which reflects the complex nature of trophic interactions within the soil food web [18,68] and challenges the assumption that biodiversity enhances ecosystem function [46]. In reality, this relationship depends on trophic position, resource background, and habitat structure [68,69].

There is a consistent positive correlation between bacterivore diversity and energy flow across all vegetation types except PT, which indicates that bacterial channels are generally diversity-dependent, and the energy transfer mediated by bacterivores is carried out through complementary resource utilization, in which higher species richness enhances the utilization of diverse bacterial prey [15]. The exception to PT may be resource constraints caused by recalcitrant litter and ECM fungi competition, which limits bacterial productivity, regardless of grazer diversity [73,78]. In contrast, the diversity–energy flow relationship (positive in CK, AV, and MS but negative in PT) of fungivores is vegetation-dependent, which shows that coniferous systems harbor a completely different fungal channel. In broadleaf and grassland systems, higher fungivore diversity increases energy flux by utilizing diverse fungal resources and niche complementation [91]; meanwhile, in the ECM-dominated coniferous system (PT, negative correlation), the increase in fungal diversity may be due to resource allocation under limited conditions rather than the enhancement of energy flow, or predation by fungivores inhibiting fungal productivity [21]. This is consistent with the result that competition between ECM fungi and saprotrophic fungi reduces the availability of overall fungal resources [78]. Similarly to the fungal channel, herbivore diversity–energy flow shows a PT-specific positive correlation. PT ‘s limited root biomass and stubborn tissue make its root environmental resources scarce, and a higher diversity of herbivores is needed to effectively utilize the available roots [16]. Omnivores–carnivores have an MS-specific and aggregate-dependent diversity–energy flow relationship (significantly positive only in MS; negative in MA, positive in larger aggregates), which shows that top-down control in grassland requires sufficient habitat connectivity to promote predator–prey interaction [9,82]. Compared with shrub- or arbor-dominated systems, grassland root architecture creates unique advantages for predator–prey interactions [17]. In MA, regardless of the diversity of predators, physical isolation may prevent them from acquiring prey [86]. However, in larger aggregates, well-connected pore networks allow different predator combinations to play an effective top-down control role, enhancing energy transfer to the upper trophic level [14].

### 4.6. Vegetation–Aggregate Interactions Shape Soil Food Web Energetics in the Loess Plateau

Our results demonstrated that vegetation type and soil aggregate size interactively determine the nematode community structure and energy dynamics. The observed vegetation–aggregate interaction patterns in energy flux and uniformity may be caused by vegetation-specific inputs (litter quality, root exudates, and symbiont associations) and aggregate-based filtering. High C:N litter from AV supported bacterial channels, while the N-rich litter from CK (due to nitrogen fixation) promoted fungal-based channels. This is consistent with the global model that litter stoichiometry is important in predicting bacterial and fungal energy pathways [17,20]. AM-associated AV can secrete activated carbon and stimulate bacterial growth and bacterivore grazing, while nitrogen-fixing CK can secrete organic acids, enhance nutrient availability, and support various fungivores and herbivores. These rhizosphere effects were particularly evident in larger aggregates, where the root density and exudate concentration were highest [16,50]. In addition, LMA (>2 mm) with high pore connectivity is conducive to bacterial energy channels and supports diverse bacterivores, while MMA (0.25–2 mm) provides high-quality habitats for fungivores and omnivores–carnivores by balancing resource availability and physical protection [10]. The physical isolation and limited resources in MA (<0.053 mm) affected all trophic groups and caused the lowest energy flux.

### 4.7. Implications for Vegetation Restoration on the Loess Plateau

The obvious functional differentiation between vegetation types in this study showed that, when developing vegetation restoration strategies for the Loess Plateau, the aboveground productivity, underground food web structure, and energy dynamics should all be taken into consideration, with direct implications for ecological restoration in this area. CK is a key restoration species in the region, which maximizes total energy flux through the soil food web, especially in SMA and MMA. At the same time, it has the ability to enhance fungal and plant-parasitic channels, maintain high energy uniformity, and support diverse nutrient interactions. Therefore, CK plantations can quickly establish a functional underground network that supports nutrient cycling and energy transfer [19,23]. This is consistent with the research results that leguminous shrubs enhance soil multifunctionality in degraded ecosystems [25,26]. Although the total energy flux of AV was lower than that of CK, it maintained an unusually high energy uniformity in MA, SMA, and MMA, indicating that its associated food web distributes energy fairly among trophic levels. This feature is conducive to enhancing the stability of the ecosystem and the ability to recover from disturbances [69]. Therefore, AV is helpful in establishing a stable and well-structured food web in ecological restoration. Here, the natural grassland SB maintains a complex, multi-channel food web with strong top-down regulation. Therefore, while achieving the goal of erosion control and carbon sequestration, the restoration strategy combining native grass with shrubs and trees is more conducive for establishing a food web structure close to the pre-disturbance state [92,93]. In addition, the strong aggregate size dependence in the energy pattern shows that soil physical structure is a key regulator of remediation results. Management practices that promote the formation of macroaggregates—such as reducing tillage, organic amendments, and maintaining plant cover—can help to optimize habitat quality and increase energy flux in different nematode communities [10,86].

Several limitations should be considered when interpreting our findings. The space-for-time substitution method used here neglected successional trajectories of food web development [3]. Long-term monitoring is needed to record temporal dynamics during vegetation restoration. The lack of historical stand data affects the understanding of the mechanisms concerning the results, and future work should include comprehensive vegetation characteristics. The inferences regarding specific mechanisms (root exudates, allelopathy, microbial community) came from the literature. Although reasonable, direct measurement of these variables in future studies will strengthen causal inference. Morphological identification is time-consuming and may lead to underestimation of cryptic species [29], while high-throughput methods lack biomass data. The integration of these two methods can simultaneously clarify the classification accuracy and realize functional quantification. The calculation of energy flux depends on the published allometric relationship and assimilation efficiency [39], which may vary with environmental conditions [44]. The sensitivity analysis confirmed that our comparative conclusions are still robust to parameter changes, but the absolute value should be interpreted cautiously. Although the observed patterns represent general trends, there is considerable variability in vegetation types and aggregation classes (as indicated by the error bars in Figures). In addition, this study only focused on nematodes, which cannot fully reflect the dynamics of soil energy flow. Future research should integrate other biological communities—including microbes, microarthropods, and earthworms—to comprehensively evaluate the energy dynamics of the multitrophic food web framework [14,45].

Despite these limitations, this study provides new insights into how vegetation types and soil aggregate structure interact to regulate the nematode community composition, energy flux, and energy uniformity in the Loess Plateau ecosystem. The clear functional differentiation between vegetation types and the dual regulatory pathways revealed by SEM promote our understanding of the mechanisms underlying soil food web energy flows and provide an empirical basis for optimizing vegetation restoration strategies to enhance the function of underground ecosystems.

## 5. Conclusions

This study demonstrated that vegetation type and soil aggregate size interactively regulate the nematode community composition, energy flux, and energy uniformity on the Loess Plateau. Vegetation type drives the functional differentiation of nematode trophic groups, where AV (AM tree) supports bacterivore dominance, CK (N-fixing shrub) enhances fungivore and herbivore energy flow, SB (native grass) sustains complex multichannel food webs, and PT (ECM conifer) suppresses nematode communities. Soil aggregates act as microhabitat filters, with MMA (0.25–2 mm) and LMA (>2 mm) serving as energy hotspots, while MA (<0.053 mm) is consistently associated with energy-poor microhabitats. Energy flux was driven by nematode abundance, while energy uniformity was regulated by diversity; soil aggregates promoted uniformity but inhibited total flux, revealing the trade-off between energy flux and distribution fairness. The diversity–energy relationship is multidirectional, trophic group-dependent, and vegetation-specific. CK and AV can improve energy flux and uniformity, respectively, and can be used as key species for vegetation restoration in this area. Our findings provide mechanistic insights into soil food web energetics and an empirical foundation for optimizing vegetation restoration strategies to enhance belowground ecosystem functioning.

## Data Availability

The original contributions presented in this study are included in the article/Supplementary Material. Further inquiries can be directed to the corresponding author.

## Supplementary Materials

The following supporting information can be downloaded at: https://www.mdpi.com/article/10.3390/microorganisms14040827/s1. Figure S1. Nematode abundance of different soil aggregates under different vegetation types. MA, SMA, MMA, and LMA are aggregates with different grain size, as detailed in Section 2. Lowercase letters denote significant differences among different grain sizes for the same vegetation type (*p* < 0.05), while uppercase letters denote significant differences between various vegetation types within the same grain size (*p* < 0.05). AV, *Prunus armeniaca* L.; PT, *Pinus tabuliformis* Carrière; CK, *Caragana korshinskii*; MS, *Medicago sativa* L.; SB, *Stipa bungeana*. Table S1. Soil nematode community abundance (%) of different soil aggregates under different vegetation types.

## Abbreviations

The following abbreviations are used in this manuscript:

CK	*Caragana korshinskii*
AV	*Prunus armeniaca* L.
PT	*Pinus tabuliformis* CarriÈRe
MS	*Medicago sativa* L.
SB	*Stipa bungeana*
LMA	Large Macroaggregates
MMA	Medium Macroaggregates
SMA	Small Macroaggregates
MA	Microaggregates
P_C_	Carbon Allocated to Production
R_C_	Total Carbon Used in Respiration
F	Energy Flux
e_a_	Assimilation Efficiency
L	Energy Loss
SI	Structural Index
EI	Enrichment Index
NMF	Nematode Metabolic Footprint
Fe	Enrichment Footprint
Fs	Structural Footprint
CFI	Comparative Fit Index
GFI	Goodness-of-Fit Index
RMSEA	Root Mean Square Error of Approximation
NFI	Normalized Fit Index
TLI	Tucker–Lewis Index
ANOVA	Analysis of Variance
HSD	Honestly Significant Difference
