# Supplementary material for: Vegetation and Soil Aggregates Shape Nematode Communities and Energy Flow on the Loess Plateau
Source: Microorganisms. 2026 Apr 3;14(4):827. doi: 10.3390/microorganisms14040827 (PMC13119492; doi:10.3390/microorganisms14040827)
Supplement: Supplementary file 1 [file microorganisms-14-00827-s001.zip › microorganisms-4178637-supplementary.pdf]

Supplementary Figure S1

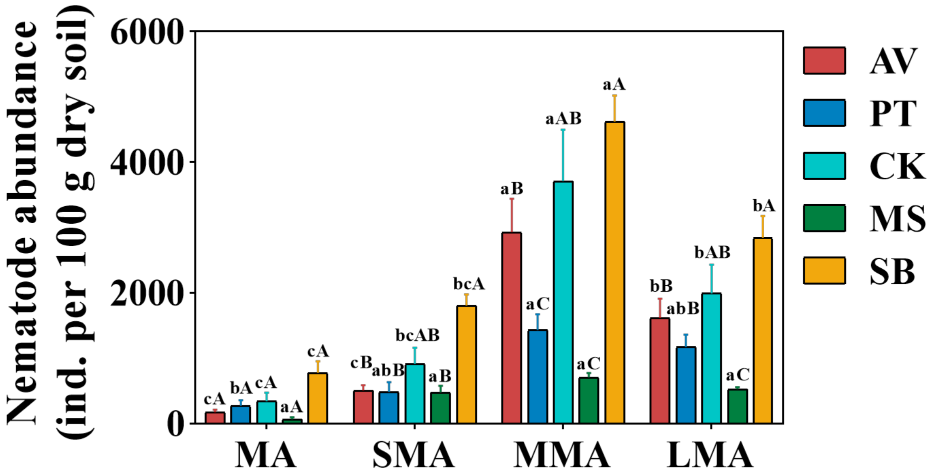

Figure S1. Nematode abundance of different soil aggregates under different vegetation types. MA, SMA, MMA, and LMA are aggregates with different grain size, as detailed in the Methods. Lowercase letters denote significant differences among different grain sizes for the same vegetation type ($P < 0.05$), while uppercase letters denote significant differences between various vegetation types within the same grain size ($P < 0.05$). AV, *Prunus armeniaca* L.; PT, *Pinus tabuliformis* Carrière; CK, *Caragana korshinskii*; MS, *Medicago sativa* L.; SB, *Stipa bungeana*.

Supplementary Table S1

Table S1. Soil nematode community abundance (%) of different soil aggregates under different vegetation types.

Genera	cp value (unit-less)	MA					SMA					MMA					LMA				
		AV	CK	PT	MS	SB	AV	CK	PT	MS	SB	AV	CK	PT	MS	SB	AV	CK	PT	MS	SB
**Bacterivores**																					
Alaimus	4	-	-	16.03	-	-	-	-	-	-	-	-	-	1.31	-	-	-	7.42	13.21	-	-
Acrobeles	2	6.53	4.07	-	0.51	-	3.57	1.81	-	-	-	9.09	2.49	-	2.65	-	19.63	9.26	2.10	11.72	1.83
Acrobeloides	2	-	-	-	-	-	-	-	-	-	-	-	-	-	-	-	2.68	0.59	-	-	-
Total		6.53	4.07	16.03	0.51	-	3.57	1.81	-	-	-	9.09	2.49	1.31	2.65	-	22.31	17.27	15.32	11.72	1.83
**Fungivores**																					
Ditylenchus	2	-	1.29	2.77	-	-	-	-	-	-	6.94	1.53	2.46	16.63	-	3.62	-	-	-	-	2.94
Filenchus	2	2.95	5.42	30.19	6.21	0.53	3.91	3.15	42.83	8.32	1.43	7.69	25.29	51.58	3.83	3.59	2.76	19.45	-	2.93	2.13
Miculenchus	2	-	-	-	-	-	-	-	-	0.75	0.71	2.82	8.31	-	2.19	-	-	-	7.34	5.86	-
Total		2.95	6.72	32.96	6.21	0.53	3.91	3.15	42.83	9.07	9.08	12.05	36.07	68.21	6.02	7.20	2.76	19.45	7.34	8.79	5.07
**Herbivores**																					
Boleodorus	2	-	-	-	-	-	-	-	-	2.31	-	-	-	8.32	6.46	11.51	3.45	-	-	5.17	-
Coslenchus	2	0.53	9.18	-	-	-	-	4.24	-	-	-	-	4.44	-	-	-	-	5.67	-	-	-
Paratylenchus	2	27.01	7.23	31.39	26.27	20.08	28.06	4.35	25.00	5.98	11.07	44.64	13.62	6.56	32.50	13.52	11.00	-	0.85	2.27	12.70
Xiphinema	5	-	15.08	-	-	-	-	22.04	-	-	-	-	-	14.44	-	-	-	-	-	-	-
Helicotylenchus	3	4.23	-	-	28.95	16.83	6.71	3.23	19.60	36.38	28.88	4.38	-	-	47.55	13.52	21.51	2.07	4.40	53.56	17.31
Rotylenchulus	3	5.02	5.06	-	4.83	-	1.71	1.02	-	-	-	1.80	1.42	-	4.51	-	10.31	5.24	-	18.72	-
Total		36.79	36.54	31.39	60.05	36.91	36.48	34.88	44.60	44.68	39.94	50.81	19.48	29.32	91.03	38.55	46.26	12.98	5.25	79.73	30.01

Supplementary table 1. Soil nematode community abundance (%) of different soil aggregates under different vegetation types (Continued)

Genera	cp value (unit-less)	MA					SMA					MMA					LMA				
		AV	CK	PT	MS	SB	AV	CK	PT	MS	SB	AV	CK	PT	MS	SB	AV	CK	PT	MS	SB
**Omnivores-carnivores**																					
Campydora	4	54.06	52.67	19.38	1.09	60.40	48.13	56.14	12.49	20.80	41.70	25.99	41.95	-	0.55	53.04	25.79	49.19	71.73	-	57.91
Ecumenicus	4	-	-	-	31.46	2.00	7.79	4.01	-	25.00	9.22	2.49	-	0.66	-	0.96	2.76	1.58	-	-	5.53
Aporcella	5	-	-	-	0.68	-	-	-	-	-	-	-	-	-	-	-	0.52	-	-	-	-
Total		54.06	52.67	19.38	33.22	62.40	55.92	60.15	12.49	45.80	50.92	28.47	41.95	0.66	0.55	54.00	29.07	50.77	71.73	-	63.43
Total		100	100	100	100	100	100	100	100	100	100	100	100	100	100	100	100	100	100	100	100

MA, SMA, MMA, and LMA are aggregates with different grain size, as detailed in the Methods. AV, *Prunus armeniaca* L.; PT, *Pinus tabuliformis* Carrière; CK, *Caragana korshinskii*; MS, *Medicago sativa* L.; SB, *Stipa bungeana*.

Supplementary Text

In order to evaluate the reliability of energy flow estimation, we use sensitivity analysis to test the influence of key parameters on total energy flow and energy flow uniformity. (1) Assimilation efficiency: The assimilation efficiency value of each trophic group was adjusted by ± 10 %, and the energy flux index was recalculated. (2) Substitution feeding preference test: in the non-uniform feeding scenario, omnivores-carnivores showed more than 20 % preference for the common bacterivores in the soil food web.
